# Cross-frequency phase synchrony around the saccade period as a correlate of perceiver's internal state

**DOI:** 10.3389/fnsys.2013.00018

**Published:** 2013-05-28

**Authors:** Chie Nakatani, Mojtaba Chehelcheraghi, Behnaz Jarrahi, Hironori Nakatani, Cees van Leeuwen

**Affiliations:** ^1^Faculty of Psychology and Educational Sciences, Laboratory for Perceptual Dynamics, University of LeuvenLeuven, Belgium; ^2^Klinik für Neurologie, Universitätsspital ZürichZurich, Switzerland; ^3^Okanoya Emotional Information Project, Exploratory Research for Advanced Technology, Japan Science and Technology AgencyWako, Japan; ^4^Emotional Information Joint Research Laboratory, RIKEN Brain Science InstituteWako, Japan

**Keywords:** EEG-eye movement co-registration, efference copy, visual tokens, up-date of visual coordinates, information processing over multiple fixations

## Abstract

In active vision, eye-movements depend on perceivers' internal state. We investigated peri-fixation brain activity for internal state-specific tagging. Human participants performed a task, in which a visual object was presented for identification in lateral visual field, to which they moved their eyes as soon as possible from a central fixation point. Next, a phrase appeared in the same location; the phrase could either be an easy or hard question about the object, answered by pressing one of two alternative response buttons, or it could be an instruction to simply press one of these two buttons. Depending on whether these messages were blocked or randomly mixed, one of two different internal states was induced: either the task was known in advance or it wasn't. Eye movements and electroencephalogram (EEG) were recorded simultaneously during task performance. Using eye-event-time-locked averaging and independent component analysis, saccade- and fixation-related components were identified. Coss-frequency phase-synchrony was observed between the alpha/beta1 ranges of fixation-related and beta2/gamma1 ranges of saccade-related activity 50 ms prior to fixation onset in the mixed-phrase condition only. We interpreted this result as evidence for internal state-specific tagging.

## Introduction

Our visual world appears stable and integrated, and yet it is largely patched together from the information acquired during single eye fixations. The eyes perform a saccadic movement followed by a fixation a few times every second; the resulting number of samples to be assembled is considerable. Are these movement all equally important, or do perceivers prioritize some samples over others?

As seen in the famous eye movement records by Yarbus (1967), the eyes do not move at random, but are directed by visual salience (e.g., Findlay and Walker, 1999; Itti and Koch, 2000; Reichle et al., 2012). On the other hand, a considerable number of fixation samples will be irrelevant and/or redundant to the current goal of visual inspection. For instance, conflicting selections may sometimes send eye movements astray (Trappenberg et al., 2001; Meeter et al., 2010; Nikolaev et al., 2011; Devue et al., 2012). Even in a simple task such as single alpha-numeric character identification, multiple fixations to a target are often made, e.g., on one third of all trials in Nakatani and van Leeuwen (2008). Of these fixations, often the first one is sufficient to perform the task. Being able to distinguish relevant from irrelevant samples would be beneficial to the efficiency of perception.

To achieve this aim, each sample perceivers expect to be relevant may be tagged with a brain signal, marking it out for later processing. Such a mechanism is more flexible than a rigorous early selection through on-line analysis of the sample, which may result in discarding information that may later be found out as relevant. Thus, early availability of a predictive signal is essential for “tagging” an upcoming fixation. Tagging, moreover, should be fast enough to keep pace with the rate of fixations. Electrophysiological markers for fixation tagging should therefore be found around saccade onset or, at the latest, in an early post-fixation period.

The efference copy of the saccade signal meets these temporal criteria—the information is available at saccade onset. The efference copy contains information, such as size, direction, and retinal coordinate of each saccade. To some degree, this information might also be obtained from the sensory feedback of extra-ocular muscle activity. For our present purpose, we cannot distinguish these two; we will address them jointly as “saccade information.” The saccade information is stochastically predictive about the upcoming fixation content. Consider the size of saccades in common, every-day settings: larger saccades are more likely between than within objects/areas. Larger saccades will therefore be more relevant for exploration than small ones; small saccades will be more relevant for detailed inspection (Unema et al., 2005; Tatler and Vincent, 2008; Graupner et al., 2011; Mills et al., 2011).

Task context will determine whether and how a predictive tag is used (Mills et al., 2011). Therefore, tagging-related brain activity would be sensitive to a manipulation on task context. In the current study, we investigated the tagging-related brain activity using EEG measurement. We recorded EEG and eye movements simultaneously from healthy human volunteers, who performed simple saccade tasks. In each of these tasks, participants were presented an object in lateral visual field, to which they were requested to make a saccade. A task instruction followed shortly after the object presentation. This involved answering an easy or a hard question about the object presented by pressing one of two alternative response buttons, or it could be an instruction to simply press one of these two buttons. Two task-conditions were introduced. In the *blocked-phrase* condition, the participant knew the type of task instruction prior to each trial, while in *mixed-phrase* condition, this was not the case. Thus, in the latter case, the participant was more uncertain about the task than in the former. Task-uncertainty was expected to increase the probability of the tagging. Due to the uncertainty, more incoming visual information might be tagged as “relevant” for later processing. Tagging-related brain activity would therefore be higher in the mixed- than the blocked-phrase conditions.

### Research strategy

To investigate saccade-tagging, some practical problems need to be addressed. Here, we describe these problems and outline our strategy to handle them.

The first problem is the reduction of electro-oculogram (EOG) artifacts without discarding signals with saccades. EOG-related activity coincides temporally with the purported brain activity relevant for saccade information. The issue of the artifact reduction is a longstanding problem in EEG data analysis; thus varieties of solutions have been proposed (see Croft and Barry, 2000, for a review). We chose Independent Component Analysis (ICA). This method has widely been applied successfully to reduce ocular artifacts in EEG data (Makeig et al., 1996; Jung et al., 2000). In the current data set, we may assume that the origin of the artifacts is different from that of brain signals, and non-normality of the artifact signal, as are required for applying the method (Hyvärinen and Oja, 2000).

The second problem is how to distinguish saccade and fixation related processes. After artifact reduction, we constructed classifiers to extract for saccade information-related and visual information processing-related brain activity. To this purpose, peri-fixation EEG was averaged. ICA was applied to the averages, so as to obtain templates for saccade-related and visual information processing-related components. Such a methods of component identification has previously been successful for peri-fixation EEG signal analysis (e.g., Kamienkowski et al., 2012). The templates were, then, applied to single trial EEG, in order to extract saccade information-related and visual information processing-related brain activities.

Third and finally, a measure for tagging-related activity needs to be determined. Saccade-related and visual processing-related information belong to different aspects of the visual system that need not always be coordinated. However, in tagging coordination between these two must be transiently established. This means that we can detect tagging activity by measuring the transient synchrony between these activities. We employed cross-frequency phase synchrony (Sauseng et al., 2008) to the classified EEG signals. The measure (cfPSI) indicates how reliable the phase relation of two oscillatory signals is over trials at a given time point. When phase synchrony is high, two signals are likely to be connected functionally. For example, Sauseng et al. (2008) reported that cfPSI between gamma (30–50 Hz) and theta (3–7 Hz) was higher for attended than unattended visual target. They interpreted the synchrony as an indication of successful memory matching between incoming visual information (gamma) and stored information (theta).

In the current study, we propose that uncertainty about the task would enhance tagging of visual information processing-related activity with saccade information-related activity. When the task context is uncertain, more information is likely to be tagged, in order to assure flexibility of later processing. We expect the tagging to be reflected in a transient coupling between the frequencies of, respectively, visual information-related and saccade-related activity components.

For the visual information processing-related activity component, the most relevant frequency band around fixation is likely to be alpha (8–12 Hz): this is the main frequency band of the Lambda complex, which reflects early visual information processing in eye-fixation related potentials (Marton and Szirtes, 1982; Kazai and Yagi, 1999). Phase locking to a fixation of ongoing alpha activity may be crucial for the emergence of the Lambda complex (Ossandon et al., 2010).

The saccade-related activity component, by contrast, may not be restricted to a specific frequency band, since saccade-related potentials typically consist of a spike in the evoked activity (Thickbroom and Mastaglia, 1985). Such a potential will appear in wide range of frequency bands of the saccade-related component. Thus, tagging would involve coupling with a wide band of the saccade-related activity. Then again, also more sustained saccade-related activity has been observed that may have its own characteristic frequency (Bellebaum et al., 2005).

## Methods

### Participants

Ten residents of Tokyo metropolitan area (Five men and five women, mean age: 22.60 year-old) volunteered to participate in the experiment. All were right-handed and had normal or corrected-to-normal vision. Participants received a remuneration of 1000 yen per hour. The research ethics committee of RIKEN had approved the experiment.

### Stimuli, task, and design

Ninety images of natural and artificial objects were rendered using a 3D object database (500 3D Object images, Volumes 1 and 2, Taschen, Köln). Natural object stimuli consisted of 45 images of animals, fish, and insects, while artificial object stimuli included 45 images of automobiles, airplanes, hand tools, and furniture. All stimuli were rendered with realistic colors and shades. All images were scaled to fit to 5 × 5° area.

**Figure 1:**
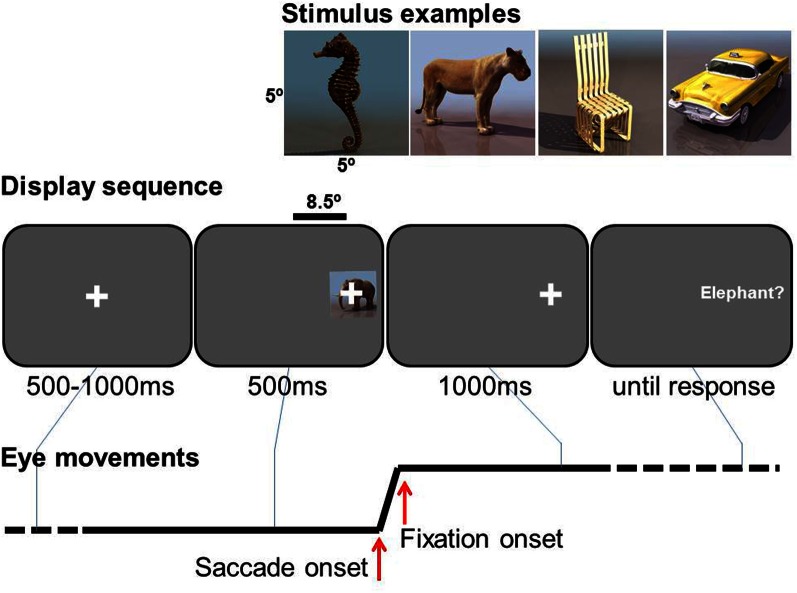
**Experimental task.** Examples of stimuli (top), a display sequence (middle, not to scale), and an example of horizontal eye movement (bottom).

The sequence of events in a trial is illustrated in Figure 1. A central fixation cross was presented for 100–500 ms, uniformly distributed in steps of 100 ms, immediately followed by object presented 8.5° either right or left, 50/50 in random order. From its onset, a small lateral fixation cross was superimposed in the center of the object as a saccade target. The object was presented for 500 ms, while the lateral fixation cross remained 1000 ms more. Participants were asked to make a saccade to the lateral fixation cross as soon as it appeared, and to keep fixating after the disappearance of the object until a phrase appeared. The phrase describes the task to be performed as either basic-level identification (e.g., “Was the image a dog?”), feature-level identification (e.g., “Was the head black?”), for which they were instructed to press the right button of a hand-held button box for “yes”, or to press left for “no”, or they were simply instructed to press the button, e.g. “Press right button.” Participants were instructed to press the button as correct and fast as possible. No response feedback was given during the trials.

In one condition the participant was informed in advance on the type of question required (basic, feature or button-press) for a following task block of 30 trials, i.e., blocked-phrase condition. The other condition was a mixed-phrase condition, in which all three types of tasks were intermixed in a pseudo-random order within each 30-trial block (10 trials for basic, feature, and button press).

### Procedure

After electrode attachment, participants were seated in a sound attenuated experimental chamber under a dimly lit condition, in front of a computer monitor used for stimulus presentation. Half of the participants started from the mixed, while the other started from the blocked conditions. Prior to each condition, 10 practice trials for each question type were given. In each condition, there were 90 trials; A half of the participants started with the mixed-phrase condition, while the other half started from the blocked-phrase condition. Within the blocked condition, the order of tasks was counterbalanced. Participants were informed about the trial type prior to each block. After completing all task blocks, the participants gave retrospective reports on their performance, and were prompted, if needed, to inform us about which condition they experienced as more “difficult.”

The task was controlled by a PC using a Visual C++ program which recorded key-press responses and generated marker signals for co-registration of eye movement and EEG recordings.

### Eye movement and EEG recordings

Binocular eye movement was recorded by a head-mounted eye tracker device (EyeLink I, SR Technologies, Ontario) with a sampling rate of 250 Hz. Calibration was performed prior to a task block, and repeated before a task when measurement error exceeded 2°. A nine point calibration pattern (center, four corners, and four mid points of four sides of the display) was used for calibrating eye-position. A drift correction procedure was used before a trial when a 1–2° error was observed. Only right-eye data were analyzed.

EEG was recorded from 14 electrodes (F3, Fz, F4, P3, Pz, P4, PO3, POz, PO4, O1, Oz, O2, HEOGs, Left VEOG) according to the international 10-10 system using differential amplifiers (Nihon Kohden MME-3132). Ag/AgCl electrodes were used for the recording. Prefrontal, central and temporal loci were not available due to the placement of the headband for the eye tracking system. Left earlobe was used as reference, and right earlobe as ground. Electrode impedance was kept under 5 kOhm. Sampling rate was 500 Hz, and low-cut 0.08 Hz and high-cut 100 Hz were applied. The data were registered separately from the eye movement and task event data. Marker signal from an independent source was sent to both systems via parallel port, which was used to align the EEG with the eye movement record off line.

### EEG data preprocessing

**Figure A1:**
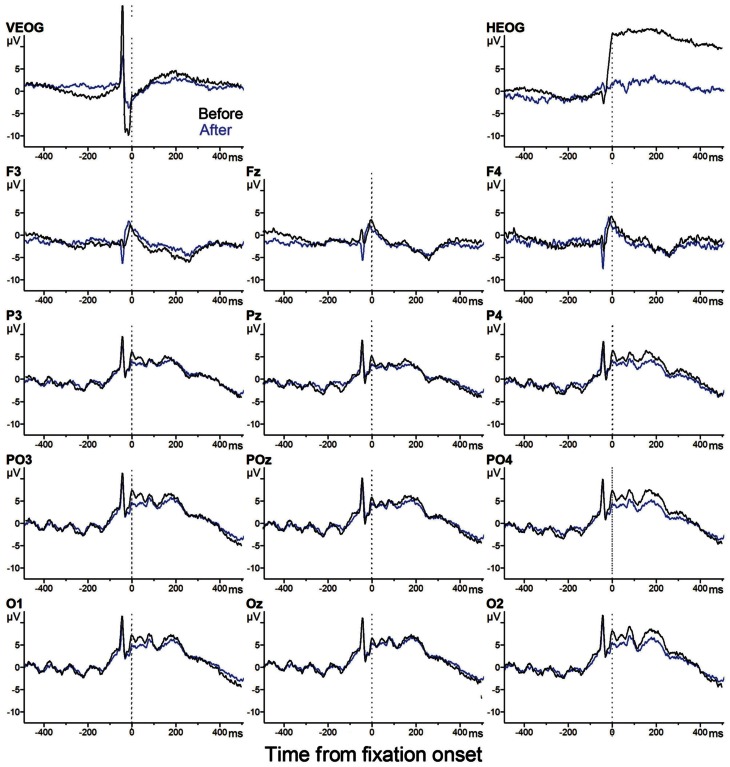
**EFRP before and after EOG artifact correction**.

For EOG artifact reduction, we applied an ICA algorithm (Hyvärinen and Oja, 1997, 2000) to the raw EEG and vertical and horizontal EOG (VEOG and HEOG) signals. For each of the 14 independent components obtained, the correlation with the EOG signals was computed. We identified as EOG components those ICA components which showed more than 70% temporal correlation with either of the EOG signals. The 70% criterion was chosen in order to balance EOG reduction and preservation of signals. In each participant, one or two EOG components were identified. These EOG components were removed before signal reconstruction. By visual inspection, we assured that the reconstructed signals showed a reduction in the VEOG and HEOG channels, while the signals in the 12 EEG channels were well-preserved (see Figure A1 of the Appendix).

The EOG artifact-reduced EEG signals was segmented into 6-s episodes (from −2500 ms to +3500 ms from fixation onset), allowing the segments to overlap. The segments were labeled by fixation type (e.g., single fixation to the stimulus or first of two fixations to the stimulus) and performance (correct response or error). Segments in error trials were excluded from further analyses together with bad eye movements (e.g., eyes moved before object onset, and blinks) and bad EEG (e.g., base-line drift and EMG/body movements detected by visual inspection) trials. In total, about 21% of all trials were discarded.

### Extraction of saccade- and visual processing-related components

**Figure 2:**
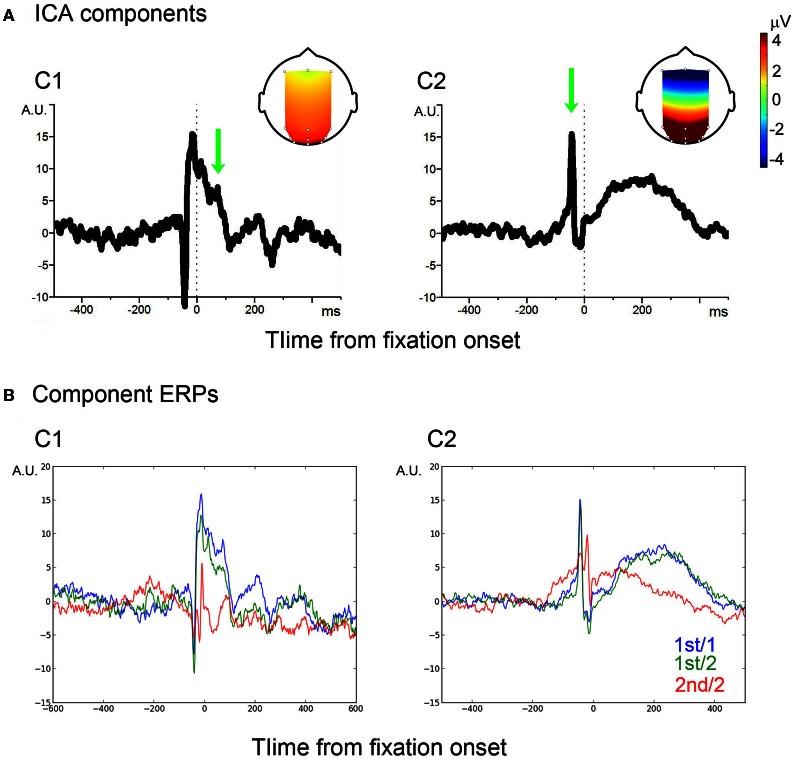
**ICA components. (A)** Two ICA components, C1 and C2. Vertical dotted-lines indicate the fixation onset. Arrows indicate P1- and SP-equivalent activities, of which the scalp projections are shown in the head image. **(B)** Average of filtered signals.

**Figure A2:**
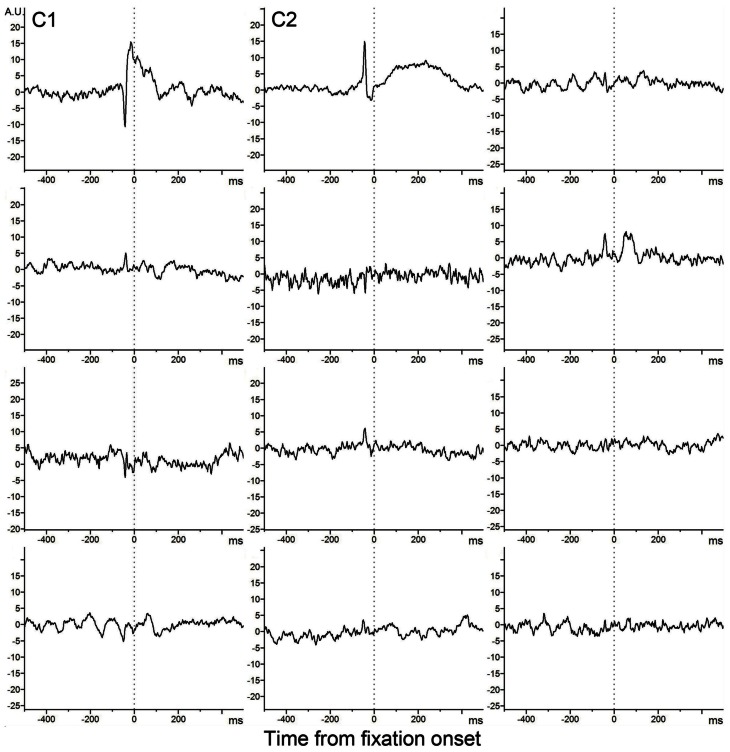
**Twelve ICA components**.

For the sake of component identification, single-fixation EEG segments of both mixed and blocked conditions for each participant were averaged, from which a grand average was computed. The grand average showed the typical peri-fixation waveform, including the spike potential (SP) and the Lambda complex (see blue traces in Figure A1 of the Appendix). To separate saccade- and visual processing-related components, InfoMax ICA (Makeig et al., 1996) was applied to the grand average. Unlike the ICA for EOG artifact reduction, only the 12 EEG channels were used. This resulted in a 12-channel × 12-independent- component forward ICA matrix. Of the 12 components, two components showed eye-fixation related activity (Figure A2 of the Appendix and Figure 2). The first component (C1) showed a sharp onset; while it corresponded to the primary saccade, this sharp onset was followed by a positive activity which showed two positive peaks around 80 and 200 ms, respectively, which are characteristic of the P1 and P2 latency of the Lambda complex (Kazai and Yagi, 2003). The topology of this component, strongest in occipital electrodes, also matches to that of the Lambda complex in previous studies. Taken together, C1 may therefore be considered as a mixture of saccade-contingent and early visual processing-related neural activity.

The second ICA component (C2) also showed a spike corresponding to the primary saccade. The spike, however, was followed by a slow wave, which peaked around 200 ms from fixation onset. The spike showed a wide scalp-distribution. The polarity was positive in occipital, occipito-parietal and parietal, but negative in frontal electrode sites. The scalp distribution and polarity match to the SP (Thickbroom and Mastaglia, 1985). The following slow wave has the same scalp distribution and polarity as the spike. A component similar to the C2 slow wave was reported in previous studies; some authors regarded the component as an EOG/eye-muscle artifact (Thickbroom and Mastaglia, 1985; Godlove, 2010); recently, however, it was associated with efference copy-based up-dating of the retinal coordinate after a saccade (Bellebaum et al., 2005). For example, patients who had suffered a focal cerebellar lesion which disabled the efference copy showed reduced amplitude in post-saccade ERPs which are compatible to the C2 slow wave (Peterburs et al., 2013). In other words, the slow wave distinguished the C2 component from an EOG artifact. Thus, the entire C2 component may be considered as a saccade-related.

The forward ICA matrix was used as classifier; the C1 classifier was a 12 × 12 matrix of which the values were zeroes except for the C1-related coefficients. The matrix was dot-multiplied with the single-trial EEG segments (12 channels by 3000 peri-fixation samples = −2500 ms to +3500 ms from fixation onset in a sampling rate of 500 Hz), which extracts the C1 contribution from the single-trial EEG segments. The C2 classifier was created and the single-trial C2 contribution was extracted likewise.

In order to assure that the classifiers effectively extracted C1 and C2 signals from single-trial EEG, the extracted C1 and C2 signals were averaged, respectively. A baseline period was chosen between −500 and −200 ms. As illustrated in Figure 2B, the average waveforms were faithful to those of the C1 and C2 templates.

### Cross frequency phase synchrony

The extracted single-trial components C1 and C2 were used for cross frequency phase synchrony analysis. cfPSI values were computed between C1 and C2 single trial signals following the procedure in Sauseng et al. (2008). Gabor expansion was applied to each single-trial component between 1 and 45 Hz using a 1-Hz step size between the center of frequencies (alpha = 0.5). This procedure estimates instantaneous phase and amplitude. The range of analysis was chosen to avoid 50 Hz AC noise and muscular activity. Arbitrary frequency pairs from C1 and C2, f_m, c1_ and f_*n*, *c*2_ (1 ≤ *m*, *n* ≤ 45), were chosen to compute the phase difference at time *t* in trial *k*, which is:

$$\Delta \Phi_{k}(f_{m,c1},f_{n,c2},t)\approx ((n+m)/2\times \text{m}\times \Phi_{k,c1}(f_{m,c1},t)-(m+n)/2\times n\times \Phi_{k,c2}(f_{n,c2},t))\text{modulus2}$$

The cfPSI in the frequency pair over the trials is defined as:

$$\text{cfPSI}\left(f_{m,c1},f_{n,c2,},t\right)=\text{abs}\left(<\text{exp}\left(j\times \Delta \Phi_{k}\left(f_{m,c1},f_{n,c2,},t\right)\right){>}_{k}\right),j=\text{sqrt}(-1).$$

The combination of 45 by 45 bins yielded 2025 frequency pairs of cfPSI in each time point.

## Results

### Task results

Percentage of correct responses were, on average, 87 and 88% for the mixed and blocked-phrase conditions, respectively. Difference between the conditions was not statistically significant, *t* < 1. On the other hand, all participants reported finding the mixed condition more “difficult” and/or “uncertain” than the blocked one. Although the percent correct did not differ, the subjective report showed that the internal states of the participants had been different between the two conditions.

### Eye movement results

Saccade and fixation parameters were computed from eye position data, using the saccade detection algorithm which is a part of the eye-tracking system. Trials were classified based on the number of saccades/fixations to the object. In single-fixation trials, only one saccade was made during stimulus presentation. In correctly answered trials, the percentage of the single-fixation trials was 41% (*n* = 621), while in 51%, a small secondary saccade was observed before target offset, i.e., two-fixation trials (*n* = 866). The ratio of single- vs. two-fixation trials was 0.72. In error trials, the ratio 0.68, was about the same, *t* < 1; multi-fixations occur in the same ratio in correct and error trials. The error trials were excluded from further analyses.

In the single-fixation trials, latency of the first-and-only (1st/1) saccade from the central fixation to the lateral object was 145 ms, saccade size was 8.41°, and duration was 45 ms on average. Eyes stayed on the object for about 300 ms. In two-fixation trials, latency, size, and duration of the first saccade (1st/2 saccade) were: 130 ms from image onset, 7.57°, and 43 ms on average, while those of the second (2nd/2) saccades were: 195 ms from 1st/2 saccade offset, 1.33°, and 17 ms, on average. The eye movement parameters within a category showed small variance; standard deviation was 0.68, 0.81, and 0.71° in saccade size, and 3, 4, and 4 ms in saccade duration, for the 1st/1, 1st/2, and 2nd/2 saccades, respectively. These saccade parameters did not differ between the blocked and mixed-phrase conditions; no paired t-test yielded *p* < 0.1. The ratio of single vs. two-fixation trials was 0.76 and 0.67 for the blocked and mixed-phrase conditions, respectively. The difference was not significant, *t* < 1. The results showed that the task conditions did not affect to saccade control.

### Cross frequency phase synchrony

**Figure 3:**
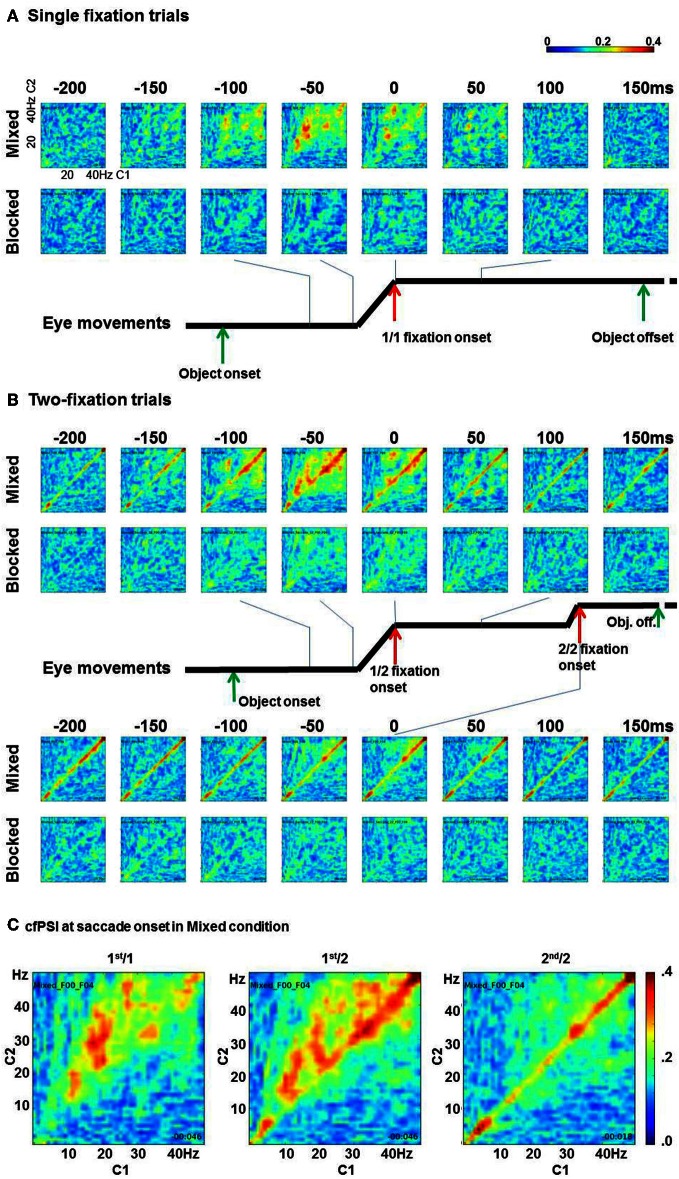
**Cross-frequency phase synchrony between C1 and C2. (A)** The cfPSI in the single-fixation trials in the mixed and blocked conditions. The x and y axes in each matrix indicate C1 and C2 frequencies. **(B)** The cfPSI in the two-fixation trials, and **(C)** The cfPSI at the onset of the 1st/1, 1st/2, and 2nd/2 saccades.

cfPSI values from all pairs were computed in all time points. Figure 3 shows eight time points round fixation onset. The x axis shows C1 frequency and the y axis shows C2 frequency. In the single-fixation trials, the cfPSI increased around saccade onset in the mixed condition. The synchrony was prominent between 10–20 Hz of C1 and 20–35 Hz of C2 activity. In contrast to the mixed condition, cross-frequency phase synchrony was not observed in the blocked condition (Figure 3A). To test the difference between conditions, the cfPSI values were averaged over 150 C1 (10–20 Hz) by C2 (20–35 Hz) frequency bin pairs and over 25 (−50 to 0 ms) time bins. The difference in the average was tested against a probability distribution generated by bootstrapping. For the bootstrapping, trials in the mixed or blocked conditions were pooled together. From this pool, *K* trials were selected allowing repeated sampling (*K* is the actual number of trials in the mixed or blocked conditions). To the re-sampled trials, the cfPSI computation and the averaging procedure were applied as in the original samples. This was repeated for 400 times to generate a probability distribution for the average difference under the null hypothesis of zero difference between the mix and blocked conditions (*H*_0_). The threshold value was 0.046 (99th percentile value, which is the upper threshold for a = 0.05 in a two-tailed test). The actual difference between conditions, 0.057, exceeded the threshold, i.e., *p*(*H*_0_) < 0.05.

The phase synchrony effect appeared when C1 and C2 showed spike-shaped activities. Frequency decomposition of spike-shaped activity may yield spurious phase-locking of oscillatory components, within as well as between C1 and C2. To check if the current effect was spurious, based on a suggestion by one of our reviewers, we reasoned that amplitude correlation should show the same pattern of results. We computed Pearson's correlations between each C1 and C2 frequency pair, following the same procedure as for cfPSI above. That is, for each participant Pearson's correlations averaged over 150 C1 (10–20 Hz) by C2 (20–35 Hz) frequency bin pairs and over 25 (−50 to 0 ms) time bins. The difference in averaged correlations between conditions was tested against the probability distribution generated by bootstrapping. The difference, 0.062, did not exceed the upper threshold value for a = 0.1, which was 0.075, i.e., *p*(*H*_0_) > 0.1. This is accordance with the waveform of the spike, which shows no visible differences in amplitude between conditions. We concluded that the difference in cfPSI was not based on an artifact.

In two-fixation trials, the cross-frequency phase synchrony also appeared higher in the mixed than in the blocked conditions (Figure 3B). Similar to the 1st/1 trials, cfPSI between C1 10–20 Hz and C2 20–35 Hz was prominent in the 1st/2 but not in the 2nd/2 saccade onset. The difference between conditions was tested using the same procedure as for the 1st/1 samples to the 1st/2 and 2nd/2 samples. For the 1st/2 samples, the difference, 0.045, exceeded the upper threshold for a = 0.05, which was 0.032, i.e., *p*(*H*_0_) < 0.05. For the cross-frequency amplitude correlation coefficient, however, the difference, −0.039, did not exceed threshold for a = 0.1, i.e., *p*(*H*_0_) > 0.1. We therefore concluded that the difference in cfPSI in the 1st/2 saccades was not attributable to an artifact. For the 2nd/2 saccades, the difference in cfPSI, 0.014, was not significant, *p*(*H*_0_) > 0.1. Neither was the difference in the amplitude correlations, 0.039, *p*(*H*_0_) > 0.1.

Specific to the two-fixation trials, phase synchrony within a band (i.e., *m* = *n*, 1:1 synchrony) was also observed. In Figure 3C, the within-band phase synchrony is prominent in 30–45 Hz and 4–8 Hz. The cfPSI of the fifteen pairs between 30 and 45 Hz were evaluated by the boot-strapping test. The difference between the mixed and blocked condition in the 1st/2 trials was 0.015, *p*(*H*_0_) < 0.05; but amplitude correlations showed the same pattern, the difference was 0.204, *p*(*H*_0_) < 0.05. Likewise, in the 2nd/2 trials the difference in cfPSI was 0.012, *p*(*H*_0_) < 0.05, but also the difference in amplitude correlation was 0.207, *p*(*H*_0_) < 0.05. The results for cfPSI within the 30–45 band, therefore, appear to be artifactual. The cfPSI of the four pairs between 4 and 8 Hz showed a difference between conditions in the 1st/2 trials of 0.039, *p*(*H*_0_) < 0.05. In the amplitude correlations, the difference was −0.118, *p*(*H*_0_) < 0.05. i.e., the correlations were *lower* in mixed than in blocked conditions. In the 2nd/2 trials, the difference in cfPSI was 0.051, *p*(*H*_0_) < 0.05, and the difference in the difference in amplitude correlations was, again, opposite: −0.132, *p*(*H*_0_) < 0.05. We concluded that the differences in cfPSI within the 4–8 bin were not artifactual. The opposite effect for the correlations is difficult to interpret. A tentative explanation is provided in the discussion.

## Discussion

Tagging of fixations could help selecting samples according to the current task context, which renders down-stream information processing more efficient. We propose that fixation tagging makes use of saccade information, and investigated its neural correlates. Reducing the EOG artifact from the peri-fixation EEG, and extracting single-trial saccade-related and visual information processing-related signals, provided data sufficient for testing our hypothesis. Phasic synchronization of the two signals was estimated using a cross-frequency phase synchrony measure (cfPSI). The results show that the synchrony increased around saccade onset in the condition where the task was designed (and reported) to have an uncertain task context.

A number of studies suggest that the most prominent electrophysiological activity of the period around the saccade is of extra-ocular muscle origin (Thickbroom and Mastaglia, 1985; Sasaki et al., 2002). The cfPSI pattern, however, is difficult to explain based on synchronization between muscular activities only. First, the saccade profiles were virtually identical in the mixed and blocked conditions. Nevertheless, cfPSI increased only in the mixed condition. Second, the frequency band of extra-ocular muscle activity spans 20–200 Hz (Kovach et al., 2011); however, the C1 band for the synchrony was 10–20 Hz, which spans the alpha and beta1 bands. Third, the cross-phase synchrony was observed before the primary, but not before the secondary fixation on the target. The classifier successfully extracted secondary saccade-contingent activities in C1 and C2, so the absence of a cfPSI effect in secondary fixations is unlikely to be due to a lack of component activity. These considerations suggest that the observed cross-frequency phase synchrony indicates coordination of visual-processing-related (C1) and saccade-related (C2) activities.

The frequency band of the C1 component overlaps in part with the alpha range (8–12 Hz), which is the main frequency of the Lambda complex. The Lambda complex reflects early visual information processing (Marton and Szirtes, 1982; Kazai and Yagi, 1999). The synchronization occurred before the onset of the Lambda complex. A contribution of pre-Lambda/ongoing alpha activity to the Lambda complex itself was reported (Ossandon et al., 2010). The C1 component extends to the beta1-band. Ito et al. (2011) reported that in monkeys, the beta1-band Local Field Potential (LFP) modulated visually induced spiking of V1 neurons. The LFP modulation was time-locked to saccade onset. Moreover, their study considered the origin of the saccade-related LFP modulation as a corollary signal.

The frequency band of the C2 component spanned beta2 to gamma1 bands. The beta2 to gamma1 band is known to modulate eye-movement, in particular saccadic reaction times (Diederich et al., 2012). Our understanding of C2 as saccade-related is based on the scalp distribution and polarity of the C2 component, which matched to the Spike Potential (Thickbroom and Mastaglia, 1985), while subsequent C2 activity corresponds to the up-date of spatial coordinates after a saccade (Bellebaum et al., 2005). The effect of task on cross-frequency synchrony, however, is limited to the SP interval. The fact that it is concentrated on the beta2 to gamma1 band may suggest, in accordance with (Diederich et al., 2012) that this activity is specifically relevant to saccade timing.

Based on these interpretations of C1 and C2, it may be possible to conceive of a function of their synchrony for impending visual information processing. We proposed that the synchrony reflects the use of efferent saccade information for tagging of fixations. Uncertainty of the task situation in our mixed condition makes fixations more likely to be tagged. According to our tagging hypothesis, efferent information is evaluated in its task context. Evaluation is reserved for a planned saccade, i.e. the primary saccade. This explains why there is no task effect for secondary saccades.

In this study, also non-cross-frequency (i.e., *m* = *n*) phase synchrony (or, in other words, conventional PSI) in the theta bands showed sensitivity to the task manipulation. This effect was observed in the two-fixation trials. It might be considered as a mechanism for organizing multiple fixations into a cognitive cluster (Graupner et al., 2011; Nikolaev et al., 2011). This remains open to investigation; another issue on which our results are inconclusive is to what extent tagging determined the fate of the fixation sample in visual encoding. A comparison between the signals on correct responses and errors could have provided this information. Unfortunately, the low numbers of errors did not permit such an evaluation of the signals.

## Conclusion

Phasic synchronization before fixation onset, between saccade-related and visual-information related activity constitutes a highly plausible neural mechanism for tagging of fixation information.

### Conflict of interest statement

The authors declare that the research was conducted in the absence of any commercial or financial relationships that could be construed as a potential conflict of interest.

## Appendix

### Effect of ocular artifact correction

Figure A1 illustrates the effect of the EOG artifact reduction (see Methods for the procedure). Eye-fixation-related potentials (EFRPs) of the 1st/1 fixation trials computed from the original EEG signals (black) and those from the signals after the artifact correction (blue) are shown. (Time 0 is the fixation onset. In practice, the EFRPs are also time-locked to the saccade onset, since the variance in saccade duration was only 3–4 ms. See Eye fixation results for more details). As expected, the artifact correction reduced horizontal and vertical EOG related signals, without distorting waveforms.

### Twelve independent components generated from 1st/1 EFRPs

Figure A2 lists all (12) independent components identified.
